# Easy, Faster, and Not Bloody: Providers' Perceptions on PrePex™ in South Africa

**DOI:** 10.1016/j.jana.2016.07.004

**Published:** 2016

**Authors:** Minja Milovanovic, Noah Taruberekera, Karin Hatzold, Neil Martinson, Limakatso Lebina

**Keywords:** device circumcision, health professionals, medical male circumcision (MMC)

## Abstract

PrePex™ (Circ MedTech Ltd., Tortola, British Virgin Islands) devices are being evaluated in several countries for scale-up of medical male circumcision (MMC) as an HIV prevention intervention. Health care workers' perceptions should be considered prior to scale-up. A cross-sectional open-ended questionnaire was administered to health care workers from nine MMC programs in South Africa that provided either PrePex™ and surgical circumcision (mixed sites) or surgical circumcision only (surgery-only sites). A total of 77 health care workers (37 at mixed sites and 40 at surgery-only sites) with median ages of 29 years (interquartile range 27-37) and 34 years (interquartile range 29-42), respectively, were recruited into the study. The perceived benefits of PrePex™ MMC for health care workers were: device simplicity, convenience, bloodless, and ease of use. Identified challenges included limited public knowledge of device, pain, smell of necrotic skin, and delayed healing. Health care providers perceived the PrePex™ MMC device to be simple and adaptable for existing MMC programs.

The World Health Organization (WHO) and Joint United Nations Program on HIV/AIDS have recommended the scale-up of medical male circumcision (MMC) for HIV prevention by using any of the three recommended surgical procedures: dorsal slit, sleeve resection, and forceps-guided methods (WHO, 2009). South African legislation has required that surgical circumcision be performed by a trained physician even though there is evidence that nurses can provide surgical circumcision as safely as physicians (Curran et al., 2011, Frajzyngier et al., 2014, World Health Organization, 2011), and there have been discussions on task shifting to allow trained nurses to perform all surgical MMC tasks (Curran et al., 2011, World Health Organization, 2013a). In contrast, MMC devices are considered to be minimally invasive, and therefore, task shifting can be implemented with no change to legislation. Devices for MMC are being evaluated in several countries as they promise to make MMC quicker, safer, more cost effective, and will not require physician providers (South African Nursing Council, 2005, World Health Organization, 2013a).

The PrePex™ (Circ MedTech Ltd., Tortola, British Virgin Islands) circumcision device was prequalified by WHO in May 2013 (WHO, 2013b); it has been evaluated for safety and efficacy and found to be a feasible option for nonphysician scale-up of MMC. The PrePex™ device consists of two plastic rings that are applied along the measured circumcision line to compress and cause distal necrosis of the foreskin, which is removed 7 days later without a need for local anesthesia or suturing (Fedlum et al., 2014). The South African Department of Health plans to circumcise 4.3 million men by 2016 (Shisana et al., 2014), but has been struggling with challenges such as overcrowding, lack of skilled personnel, poor infrastructure, and not enough physicians in health facilities (Keeton, 2010, World Health Organization, 2000).

As innovative methods are evaluated for MMC scale-up, the perspectives of relevant health care workers should be considered (WHO, 2011). Health care workers act as important sources of health advice and medical information for the public, and their buy-in is important for the rollout of any new circumcision methods (Mavhu et al., 2014, Naidoo et al., 2012).

The objective of our study was to assess PrePex™ MMC device perceptions, attitudes, user experiences, and training requirements of health care workers who work in mixed (surgery and PrePex™) MMC sites (mixed-site providers) or surgical MMC only (surgery-only providers).

## Methods

A cross-sectional survey of perceptions, attitudes, user experiences, and training requirements of the PrePex™ MMC device was conducted from October to December 2013 in nine MMC clinics across four provinces (Gauteng, Mpumalanga, Free State, and North West) in South Africa. To be eligible for the study, a health care worker had to have some experience working with MMC, and performed or observed PrePex™ MMC. We approached health care workers (physicians, clinical associates, nurses, counselors, and managers) who worked in mixed sites (PrePex™ and surgical) and surgery-only sites to answer a self-administered questionnaire. All health workers were considered for inclusion as they all interacted with patients and provided MMC services. Surgery-only sites answered the questionnaire after attending an information session (demonstration videos and posters) on PrePex™ MMC because they had not previously observed the PrePex™ MMC procedure. Participants working in mixed sites had either done the PrePex™ device procedure or observed PrePex™ circumcision; therefore, no additional information session was required prior to administering the questionnaire. Two different questionnaires for mixed sites and surgery-only sites, with open- and closed-ended questions, were used. All participants were reimbursed ZAR25 (∼USD 2) for taking part in the study.

### Setting

The study was conducted in nine MMC clinics. Eight circumcision clinics were high-volume MMC sites (800-1,000 circumcisions a month capacity) and one was a low-volume MMC site in an HIV wellness clinic. All nine sites performed surgical circumcision (mainly forceps guided) using single-use prepackaged surgical kits following the MOVE Model (WHO, 2010), prior to the introduction of PrePex™. During the study, three clinics (Witbank, Tsakane, Zuzimpilo) were mixed sites that provided both PrePex™ and surgical circumcision. Across these three sites, nine health care workers were trained as PrePex™ providers: three medical officers, three clinical associates, and three professional nurses. The remaining clinic staffs at these three mixed sites were trained to assist PrePex™ providers with the MMC procedure, and the counselors were trained to provide appropriate information on PrePex™ to potential participants. More information on the mixed sites has been provided by Lebina and colleagues (2015). The other six MMC sites (Pelonomi, Tshepong, Potchefstroom, Botshabelo, Piet Retief, Khula Ndoda) provided surgical MMC only.

### Study Procedure

Following PrePex™ MMC demonstration, staff individually consented to take part in the study and then completed a self-administered questionnaire in English. In the mixed sites we collected information on participant demographics, training, user experience, opinions on community acceptability, how it compared to surgical MMC, procedure preferences, and recommendation perceptions. In the surgery-only sites, the survey collected information on participant demographics, perceptions of training required to use the PrePex™ MMC device, opinions on community acceptability, and how it compared to surgical MMC procedure preferences. Participants were encouraged to complete the questionnaire based on their own views of the PrePex™ device, the procedure, and experiences using the PrePex™ device or working in an MMC clinic. Most of the questions were open-ended, such as: *When you first heard about PrePex™, what did you think?* and *In your opinion, what are some of the disadvantages of using the PrePex™ circumcision device?* Some of the closed-ended questions were: *Do you think male circumcision using the PrePex™ device is something that can be introduced into South African communities?,* and *age, gender, race, position at clinic,* and *where were you trained?*

### Data Analysis

Participant responses were collected and transcribed in order to demonstrate attitudes and perceptions about PrePex™ MMC. Braun and Clarke's (2006) six steps of thematic analysis were used to analyze the findings by coding participant responses. This method allowed for the categorization of patterns of data and insight into individual and collective perceptions of PrePex™ device circumcision. Quantitative data were analyzed descriptively using SPSS version 22 (IBM, Armonk, NY) and were used to support qualitative findings.

### Ethics Statement

All participants provided written informed consent and the University of the Witwatersrand Human Research Ethics committee approved the study.

## Results

A total of 77 out of 99 health care workers participated in the study during the 3-month study period. Of the remaining 22, three were not eligible for the study and 19 were not on site when the questionnaires were administered. Of the total recruited, 37 were mixed-site providers and 40 were surgery-only providers.

The median age for the mixed-site providers was 29 years (interquartile range 27-37) and for surgery-only providers was 34 years (interquartile range 29-42). The majority (Table 1) of health care workers in both groups were female (73% of mixed-site providers and 75% surgery-only providers) and nurses (54% of mixed-site providers and 71% of surgery-only providers). On average, mixed-site providers had been working with PrePex™ for 3 months at the time of the study. A total of three main themes (with sub-themes) on provider acceptability were identified: perceived PrePex™ benefits, health care worker concerns about the PrePex™ device, and training requirements.

### Perceived PrePex™ Benefits

The benefits of PrePex™ were categorized into three sub-themes: ease of procedure, perceived benefits to clients, and medical and traditional considerations.

#### Ease of procedure

Health care workers were pleased with the device and said that it seemed user friendly, simple, convenient, bloodless, and easy to maintain: “simple and quick” (Nurse, surgery-only site). Health care workers also thought that PrePex™ circumcision would be quicker than surgical circumcision, and therefore, one health care worker could do more circumcisions and the program could easily be scaled up, “… may be good for peak seasons when we have high volume of clients” (Nurse, surgery-only site). PrePex™ MMC was considered to be more cost-effective because anesthesia and sutures were not required, less consumables and instruments were used, and little medical waste was produced: “The cost seems less with PrePex™ …” (Management, mixed site); “… it save(s) equipment and drugs” (Nurse, surgery-only site). Significantly more (*p* < .001) mixed-site providers (89%) compared to surgery-only providers (40%) preferred PrePex™ MMC over surgical circumcision (Table 2).

#### Perceived benefits to clients

This sub-theme included noninvasiveness, no bleeding, less anxiety, and a higher uptake of MMC. Mixed-site providers perceived that clients could return to daily activities sooner, and 76% thought PrePex™ would have fewer adverse events. Respondents suggested that wound care would be easy to maintain. Thirty-five percent of mixed-site providers acknowledged that their perceptions of PrePex™ changed once they started working with it; “From skeptical to now I'd vouch for it. Good idea. Results are very good” (Physician, mixed site).

#### Medical and traditional considerations

Mixed-site providers saw PrePex™ as a solution to existing challenges such as task shifting because PrePex™ circumcision could be performed by nurses. The minimal use of blades and local anesthetic was seen as a positive to reduce the risk of sharps injuries. There were also thoughts from surgery-only providers that traditional circumcision providers could be easily trained and possibly bridge the gap between medical and traditional circumcision, leading to safer traditional circumcision, “Traditional circumcision leaders can be trained and certified to do the procedure, thus diminishing a high rate of sepsis and death” (Nurse, surgery-only site).

Both mixed-site providers (100%) and surgery-only providers (88%) indicated that the PrePex™ device could be incorporated into existing MMC programs in South Africa with additional patient education and training for health care workers.

### Health Care Worker Concerns about PrePex™ Device

Health care workers perceived challenges with PrePex™ that could affect rollout. These challenges included novelty, smell of necrotic foreskin, trusting the patient, no cutting, and the role of health care workers.

Participants thought that the novel PrePex™ device might not be easily accepted by people who believed that circumcision should be painful.

Good initially, however it will be hard to attract more clients because the forceps guided was the closest to what was done on the mountains culturally, which bears a pride of a manhood cut. It is a modern and more improved tool but does not suit the cultural specific of the target group … (Clinical Associate, surgery-only site)

Surgery-only site providers were also concerned that PrePex™ might be confused with the Tara Klamp (which had received negative publicity) and instruments used on farm animals for castration: “This could be a challenge since African communities use almost same method for their animals. So they may be doubtful about the method” (Physician, surgery-only site). Furthermore, mixed-site providers thought the smell of necrotic foreskin was a possible deterrent.

Health care workers noted that in order for PrePex™ to work, the client would have to comply with instructions (to elevate the penis, not rub or remove the device, abstain from sexual intercourse and masturbation, and return for follow-up clinic visits if there were problems) given at MMC clinics: “it takes too long to heal and can lead to sex temptations” (Nurse, surgery-only site).

Other concerns of both mixed sites and surgery-only sites were that some patients had to be excluded due to anatomical abnormalities such as phimosis and tight foreskin and that the device came in various sizes and it would be the providers' responsibility to determine the appropriate size. Furthermore, the fact that one person could do more circumcisions also made surgery-only providers worry about job security. The task shifting to mainly nurses might be opposed by traditional circumcision providers who find women's involvement in male circumcision culturally inappropriate.

### Training Requirements

Mixed-site providers considered the training provided (1 day of theory, 15 PrePex™ placements, and 10 removals) to be sufficient to make them competent with the PrePex™ MMC: “At the end of the training I was comfortable to screen, place, and remove” (Management, mixed site). However, some indicated that four placements by trained surgical MMC providers would have been adequate. Therefore, PrePex™ training could be designed according to the level of health care worker experience in MMC.

The surgery-only providers also said that PrePex™ was an easy-to-learn technique that could be learned in 4 days by both nurses and physicians, “After we gain some experience we can do it because [it] is a simple procedure” (Nurse, surgery-only site).

## Discussion

Our study demonstrated that the PrePex™ MMC device was considered by health care workers to be easy, simple, quick, and convenient, and could be incorporated into existing MMC programs. Previous observations in other studies also indicated that device MMC was the preferred option for clinicians and that they would recommend it to others (Fedlum et al., 2014, Kigozi et al., 2013, Sokal et al., 2014).

Scale-up of MMC has been limited by the availability of providers (Mavhu et al., 2014, Samuelson et al., 2013); therefore, it is possible that PrePex™ could improve MMC scale-up with shorter procedure times and using nurses to place the device (Mutabazi et al., 2013a), especially with seasonal fluctuation of demand (higher only in winter months; de Bruyn et al., 2007). Nurses were receptive to and comfortable with the PrePex™ MMC device and procedure, which could make MMC task shifting from physicians to nurses easier.

Fear of perceived pain has been regarded as a barrier for MMC access (Evans et al., 2014, Sahay et al., 2014). Communicated messages about minimal pain with PrePex™ MMC could discourage traditional men who believe in painful circumcision, although pain has been regarded as a barrier to MMC.

The health care workers' opinions that PrePex™ was less expensive than surgical MMC has been observed in Ugandan and Rwandan studies where PrePex™ cost 2% to 36% less than surgical MMC (Duffy et al., 2013, Mutabazi et al., 2013b). However, a cost evaluation in this setting has shown that PrePex™ and surgical circumcision costs were similar at a mixed site (USD $59.53 and $59.62), and cost reduction could only be observed in a PrePex™-only site (Kim et al., 2015).

In some studies, clients reported a quicker resumption of normal activities after PrePex™ compared to surgical circumcision (Fedlum et al., 2014), as was also noted in our study, although healing time was longer with PrePex™ (Mutabazi et al., 2012, Mutabazi et al., 2013a).

Health care workers are at risk for blood-borne infections (Adefolalu, 2014), and there is no postexposure prophylaxis at some mixed sites (Rech et al., 2014), which could explain why no needles or suturing was perceived as a benefit with PrePex™.

Early sexual resumption and device displacements have been reported in other studies, and this reinforced the concern that health care workers had about trusting men to follow instructions (Fedlum et al., 2014, Hwett et al., 2012, Lebina et al., 2015, Mutabazi et al., 2013a).

It was not clear why the clinical staff were concerned with potential job losses due to PrePex™ implementation when there were numerous vacancies in health departments across the country (Gauteng Provincial Government, 2013).

Little information has been published on health care workers' perceptions on the training requirements for providing PrePex™ device circumcision. Previous PrePex™ studies had noted that staff were easily trained to use the device and perform the procedure, with staff becoming more confident and exhibiting improved procedure times by day 4 of training (Duffy et al., 2013) as observed by the trainers. Similarly, in our study, health care workers also reported improved confidence and procedure times relative to the number of PrePex™ device MMC procedures performed.

### Nursing Implications

Nurses comprise the largest group of health care workers across MMC sites surveyed in our study (Table 1). However, current South African legislation has not allowed nurses to perform surgical circumcision. Nurses in our study indicated that they considered the PrePex™ procedure to be simple and something that could be learned in a short period of time. Furthermore, nurses who had worked with PrePex™ were comfortable using the device. This was significant in that task shifting from physicians to nurses was possible, which could improve MMC scale-up with shorter procedure times, especially during the seasonal fluctuation of demand.

### Study Limitations

Our study was conducted in MMC clinics with experienced MMC staff who already believed that scaling-up circumcision was important for South Africa. In order to fully explore perceptions and acceptability of the PrePex™ device and procedure in health care providers, it would be important to extend the investigation to providers not as experienced in MMC. The surgery-only providers had limited exposure to the PrePex™ device and procedure; their primary introduction was through an information session related to our study. It would be important to see how perceptions of PrePex™ change if a surgery-only provider is given an opportunity to observe a PrePex™ MMC procedure in person.

## Conclusion

PrePex™-based circumcision has been shown to be highly acceptable, with a change in PrePex™ perceptions after use from 60% to 90% in favor (Duffy et al., 2013, Galukande et al., 2014). This was similar to our findings in which almost all mixed-site providers became more accepting of PrePex™ after having worked with it. Among surgery-site health care workers who had never worked with PrePex™ device MMC, a few were hesitant to recommend including PrePex™ in the MMC program in South Africa despite having similar perceptions about benefits and challenges as PrePex™ providers. Perceived cultural challenges will need to be addressed, with appropriate communication interventions to increase acceptability and compliance. Nurses were comfortable to be PrePex™ MMC device providers. In conclusion, the PrePex™ device and procedure was perceived to be acceptable by most health care workers, but training requirements and concerns regarding potential challenges must be taken into consideration prior to rollout.

## Figures and Tables

**Table 1:** Select Baseline Characteristics of Both Groups of Health Care Workers Participating in the Study

	PrePex™ and Surgical MMC providers (Mixed Site)	Surgical MMC Providers Only^a^ (Surgery-Only Site)
Total number recruited	37	40
Median age (IQR)	29 (27-37)	34 (29-42)
Gender		
Male % (*n*)	27% (10/37)	25% (10/40)
Female % (*n*)	73% (27/37)	75% (30/40)
Position at clinic		
Physician % (*n*)	8% (3/37)	13% (5/38)
Clinical Associate % (*n*)	3% (1/37)	5% (2/38)
Nurse % (*n*)	54% (20/37)	71% (27/38)
Counselor % (*n*)	19% (7/37)	3% (1/38)
Management % (*n*)	8% (3/37)	8% (3/38)
Other % (*n*)	8% (3/37)	

*Note.* MMC = medical male circumcision; IQR = interquartile range.

a Two surgery-only providers did not indicate their positions in the clinic.

**Table 2:** Health Care Workers' MMC Method Preferences

	Health Care Workers Using PrePex™ Device^a^ (Mixed Site)	Health Care Workers Not Using PrePex™ Device^b^ (Surgery-Only Site)
Forceps guided	5.4% (2/37)	47.5% (19/40)
PrePex™	89.1% (33/37)	40% (16/40)
Other	5.4% (2/37)	12.5% (5/40)

*Note.* MMC = medical male circumcision.

a One person did not answer the question and one participant stated that their choice would depend on circumstance.

b Due to lack of experience with PrePex™, some participants did not feel that they could provide a response.
